# Molecular Docking of SP40 Peptide towards Cellular Receptors for Enterovirus 71 (EV-A71)

**DOI:** 10.3390/molecules26216576

**Published:** 2021-10-30

**Authors:** Malihe Masomian, Salima Lalani, Chit Laa Poh

**Affiliations:** Centre of Virus and Vaccine Research, School of Medical and Life Science, Sunway University, Bandar Sunway, Petaling Jaya 47500, Selangor, Malaysia; salima.l@imail.sunway.edu.my

**Keywords:** hand, foot and mouth disease, molecular docking, receptor blocking, nucleolin, SCARB2, annexin2, human tryptophanyl-tRNA synthetase

## Abstract

Enterovirus 71 (EV-A71) is one of the predominant etiological agents of hand, foot and mouth disease (HMFD), which can cause severe central nervous system infections in young children. There is no clinically approved vaccine or antiviral agent against HFMD. The SP40 peptide, derived from the VP1 capsid of EV-A71, was reported to be a promising antiviral peptide that targeted the host receptor(s) involved in viral attachment or entry. So far, the mechanism of action of SP40 peptide is unknown. In this study, interactions between ten reported cell receptors of EV-A71 and the antiviral SP40 peptide were evaluated through molecular docking simulations, followed by in vitro receptor blocking with specific antibodies. The preferable binding region of each receptor to SP40 was predicted by global docking using HPEPDOCK and the cell receptor-SP40 peptide complexes were refined using FlexPepDock. Local molecular docking using GOLD (Genetic Optimization for Ligand Docking) showed that the SP40 peptide had the highest binding score to nucleolin followed by annexin A2, SCARB2 and human tryptophanyl-tRNA synthetase. The average GoldScore for 5 top-scoring models of human cyclophilin, fibronectin, human galectin, DC-SIGN and vimentin were almost similar. Analysis of the nucleolin-SP40 peptide complex showed that SP40 peptide binds to the RNA binding domains (RBDs) of nucleolin. Furthermore, receptor blocking by specific monoclonal antibody was performed for seven cell receptors of EV-A71 and the results showed that the blocking of nucleolin by anti-nucleolin alone conferred a 93% reduction in viral infectivity. Maximum viral inhibition (99.5%) occurred when SCARB2 was concurrently blocked with anti-SCARB2 and the SP40 peptide. This is the first report to reveal the mechanism of action of SP40 peptide in silico through molecular docking analysis. This study provides information on the possible binding site of SP40 peptide to EV-A71 cellular receptors. Such information could be useful to further validate the interaction of the SP40 peptide with nucleolin by site-directed mutagenesis of the nucleolin binding site.

## 1. Introduction

Enterovirus 71 (EV-A71) belongs to the Enterovirus A species of the Picornaviridae family and is known to cause hand, foot and mouth disease (HMFD). It poses significant health risks to young children under 5 years of age and the infection has also been reported in older children and adults [1]. HFMD has become a major public health problem as it was associated with several large-scale outbreaks in the Asia Pacific region [2]. There were significant morbidity and mortality in HFMD outbreaks from Taiwan and China [3,4,5]. HFMD is generally a mild and self-limiting disease characterized by fever, rashes on the hands and feet, and mouth ulcers. Complications of HFMD infections include aseptic meningitis, brainstem encephalitis, acute flaccid paralysis and pulmonary edema which could lead to death [1,6,7]. In 2017, almost two million cases of HFMD, including 96 deaths, were recorded in China. Less than a year later, in July 2018, 1,381,685 cases of HFMD were reported, including 26 fatal cases [8]. The major pathogens involved in the majority of HFMD outbreaks were EV-A71 and CV-A16, but recent studies showed that, besides these two pathogens, other enteroviruses, such as CV-A6 and CV-A10, have emerged as significant pathogens from recent HFMD outbreaks [9,10,11]. 

To date, three formalin-inactivated monovalent EV-A71 vaccines have been approved by the China Food and Drug Administration (CFDA) [12]. However, multiple boosters (every 6–12 months) are needed to elicit protective immunity against EV-A71 and there is no cross-protection against other enteroviruses [13]. Besides vaccines, an alternative approach is to develop antiviral agents for the treatment of HFMD by targeting the virus or the host cell. Since virus replication is dependent on the host cell, there is a need to select a target so that an effective and safe antiviral drug can be designed without harming the host. Most antiviral drugs are small-molecule inhibitors that target different stages of the viral life cycle by interacting with a virus or host proteins which are critical for attachment and virus replication [14]. However, continuous mutations could occur in the virus genome, frequently leading to resistance to small-molecule antiviral drugs [15]. Developing peptides as antivirals, targeting either the virus or the host cell, could be effective strategies. The virus life cycle, comprising various stages, such as virus entry, virus replication and assembly, have been targeted for new antiviral discovery [16]. Since peptides can disrupt protein–protein interactions, they are effective as antivirals to inhibit receptor interaction with the virus. 

Enterovirus 71 is a single-stranded positive-sense RNA virus that relies on its VP1 capsid protein to attach to target cells. To date, several receptors have been proposed to be involved in EV-A71 attachment and entry, such as human scavenger receptor class B member 2 (SCARB2) [17], P-selectin glycoprotein ligand-1 (PSGL-1) [18], heparan sulfate (HS) proteoglycan [19], annexin A2 [20], nucleolin [21], dendritic cell-specific intercellular adhesion molecule-3-grabbing non-integrin (DC-SIGN) [22], fibronectin [23], human tryptophanyl-tRNA synthetase (hWARS) [24], vimentin [25], human prohibitin (PHB) [26], human galectin-1 [27], human cyclophilin [28], and sialic acid [29]. SCARB2 is the only receptor that had been reported to be involved in the attachment and internalization of the virus following uncoating [30]. Besides, SCARB2, human cyclophilin was reported to be involved in entry and enhancing the uncoating of EV-A71virion [28]. For ease of reading, we have collectively referred to them as receptors.

A synthetic peptide, SP40 (15-mers, QMRRKVELFTYMRFD), derived from the VP1 capsid of EV-A71 was shown to be a potential antiviral for inhibition of EV-A71 in human Rhabdomyosarcoma (RD) cells. SP40 could reduce cytopathic effects in RD cells caused by EV-A71 strains from genotypes A, B and C, with an IC_50_ ranging from 6–9.3 µM. The in vitro inhibitory study of SP40 showed that it significantly reduced the formation of infectious viral particles, total viral RNA and VP1 protein. Besides EV-A71, SP40 also exhibited broad-spectrum antiviral activities against CV-A16 and poliovirus type 1 in RD cells [31]. Studies of the mechanism of action suggested that the SP40 peptide was not virucidal and it probably blocked viral attachment to the RD cells through direct interaction with the cell receptor(s). SP40 might inhibit virus infection by attaching to one or more of the cell receptors. Interaction of the SP40 peptide with any of the attachment receptors could lead to inhibition of viral attachment and reduced EV-A71 infection [31]. So far, there is no available information on the attachment mechanism of the SP40 peptide to cell receptor(s). This study aims to determine the interaction site of the RD cells receptor(s) with SP40 peptide by in silico study through molecular docking analysis, followed by receptor blocking using specific antibodies for selected receptors. 

Due to a great deal of interest in therapeutic peptides, several protein-peptide docking techniques have been developed which lead the study and optimization for drug screening and design. The protein-peptide docking algorithms can be divided into two classes: (1) template-based docking, which uses structural data from the analogous protein-peptide complex, and (2) template-free docking that does not require any template and is subdivided into global and local docking. A combination of global and local docking approaches could provide a different level of prediction accuracy and is determined by the amount of information supplied as an input for the interactions [32]. Global docking is performed via a coupled search for the peptide binding site and pose. In local docking, the search for peptide-bound conformation is limited to the vicinity of the expected binding site. There are several software packages developed for protein-peptide docking, such as GalaxyPepDock [33], MDockPeP [34], HPEPDOCK [35], CABS-dock [36], pepATTRACT [37], DINC [38], AutoDock CrankPep (ADCP) [39], HADDOCK peptide docking [40] and GOLD (Genetic Optimization for Ligand Docking) [41]. The performances of these softwares have been evaluated using an extensive benchmark data set involving 185 complexes with peptide length ranging from 5 to 20 residues by Weng et al. [42]. The study revealed that, in global docking, HPEPDOCK performed the best on the entire data set and in local docking, GOLD achieved the best performance based on the peptide length of 11–15 residues.

HEPEDOCK is a webserver designed for blind protein-peptide docking through a hierarchical algorithm [35]. It considers the peptide flexibility through an ensemble of peptide conformations generated by the MODPEP program [43]. Global peptide docking is much more challenging due to the relatively larger search space compared to local peptide docking. Blind peptide-docking algorithms, such as CABS-dock [36], pepATTRACT [37] and MDockPeP [34] required lengthy simulations in peptide binding refinement and mostly takes hours on a GPU or multi-core CPUs for docking a peptide. Among them, the pepATTRACT web server is fast, but peptide flexibility is not sufficiently considered because it only considers three idealized conformations for a peptide [44] while HPEPDOCK adequately considers peptide flexibility by generating a large number of peptide conformations [35].

Unlike the good progress that can be made in docking small organic molecules to protein targets, the prediction of the complex structure between a peptide ligand and protein receptor is not easy because of the flexible nature of peptides. The Rosetta FlexPepDock web server provides an interface to high-resolution peptide docking (refinement) protocol for the modeling of peptide–protein complexes implemented within the Rosetta framework [45]. Starting from a coarse model of interaction, FlexPepDock performs a Monte Carlo Minimization-based approach to refine all degrees of freedom of peptides (rigid body orientation, backbone and side-chain flexibility) as well as protein receptor side chains conformations.

GOLD is an automated ligand docking program for local docking that uses a genetic algorithm to explore the full range of ligand conformational flexibility with the partial flexibility of the protein in the vicinity of the protein active site [41]. “Goldscore-CS is the original scoring function that is made up of four components; protein-ligand hydrogen bond energy (external H-bond), protein-ligand van der Waals (vdW) energy (external vdW), ligand internal vdW energy (internal vdW), and ligand torsional strain energy (internal torsion)”. In Goldscore, the higher fitness representing a higher affinity to the ligand.

In this work, we present a screening strategy dedicated to the predictions for the cell receptor-SP40 peptide complexes using HPEPDOCK global docking tool [35] and high-resolution FlexPepDock refinement and scoring [45] of the protein–peptide complex. During HPEPDOCK docking simulation, the search for the receptor–peptide interaction interface covered the whole surface of the cell receptors. Subsequently, the top five refined receptors-SP40 peptide complexes were analyzed using GOLD software to search for the receptors with the highest binding score. In all steps, the 3D structure of cell receptors or the receptor-SP40 peptide complexes were energy minimized using the YASARA software. Furthermore, validation, based on specific antibody inhibition, was performed to rationalize the analysis of molecular docking of SP40 peptide with EV-A71 receptors. 

## 2. Results and Discussion

### 2.1. Global Docking to Identify the Binding Site of Receptors to the SP40 Peptide

**Figure 1:**
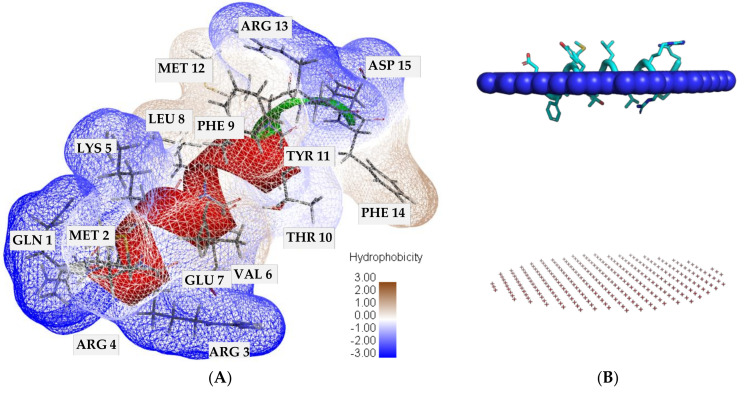
Analysis of the predicted SP40 peptide. (**A**) Hydrophobic surface area. (**B**) Ability of SP40 peptide to passively penetrate across the lipid bilayer.

The synthetic SP40 peptide comprising 15 amino acids, QMRRKVELFTYMRFD, was identified as an antiviral agent against EV-A71 infection through the screening of overlapping peptides covering VP1 [31]. The SP40 peptide has a molecular weight of 2.02 kDa with an isoelectric point of 9.98, an aliphatic index of 45.33 and grand average hydropathicity (GRAVY) of −0.833 (analyzed by ExPASy-ProtParam tool (https://web.expasy.org/protparam/) (accessed on 2 March 2021). The GRAVY value represents the hydrophobicity of a peptide and a positive GRAVY value indicates hydrophobicity, while the negative value indicates that the peptide is hydrophilic. The predicted three-dimensional structure of the SP40 peptide is a short α-helix. The hydrophobic surface area of the SP40 peptide visualized by Discovery studio software is shown in Figure 1A. 

The three-dimensional structure of the SP40 peptide has a molecular surface volume of 1909.25 Å^3^ and a molecular surface area of 1347.50 Å^2^ (analyzed by YASARA software). Analysis of the SP40 peptide by CELLPM (Figure 1B), a web-based tool aimed at validating the ability of peptides to passively penetrate across the lipid bilayer, showed that its log permeability coefficient is −30.17 [46]. Values of logP_calc_ > −5 indicate that the peptide might readily penetrate across the lipid bilayer. In wet-lab experimentation, the fluorescein-labeled SP40 peptide viewed with fluorescent microscopy showed that the SP40 peptide crossed the cell membrane and accumulated within the RD cells (unpublished observation).

In the first step, molecular docking of the SP40 peptide to target receptors of EV-A71 was carried out using HPEPDOCK to identify the binding site of SP40 peptide in each reported cell receptor. The peptide flexibility was considered by generating an ensemble of 1000 peptide conformations. The sampled peptide conformations were globally docked against the whole protein using the MDock rigid docking protocol. The top 100 peptide binding models and the docking scores and rankings for the top 10 binding models were saved and presented (Supplementary Table S1). The docking scores provided by HPEPDOCK did not reflect the real binding affinities, but a relative ranking among different binding models. 

**Figure 2:**
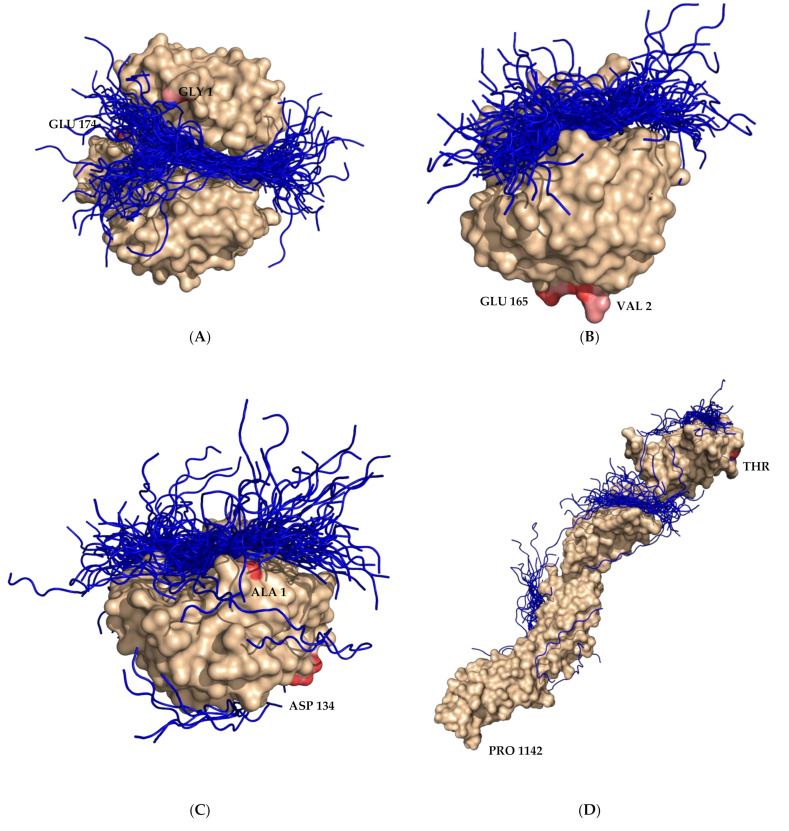

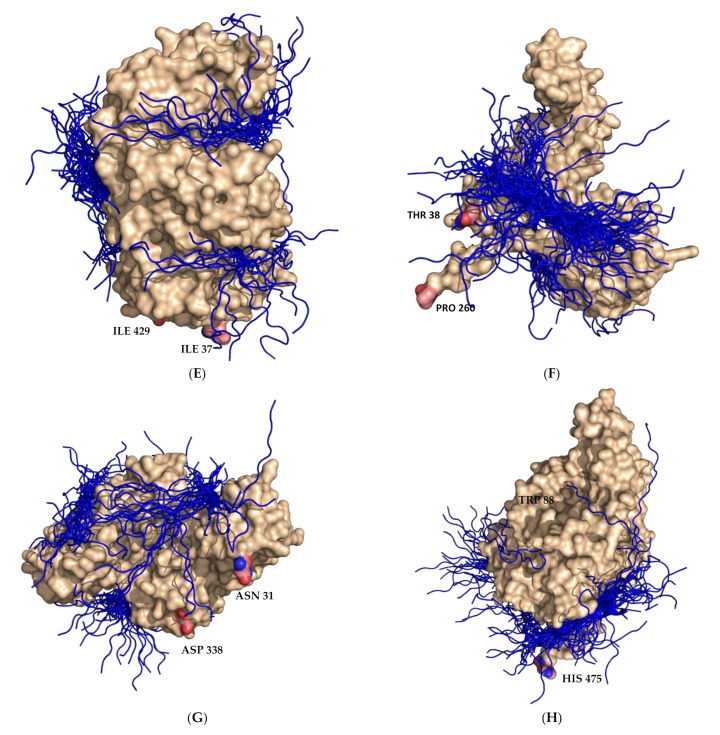

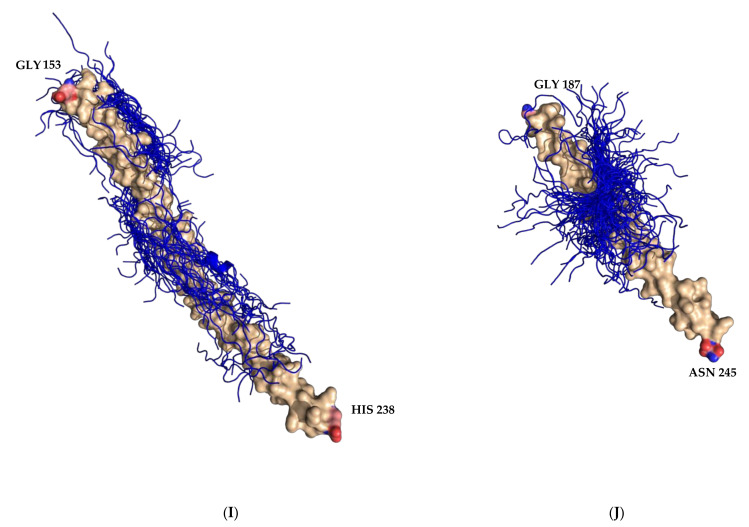
Generation of receptor SP40 peptide complex models resulting from global molecular docking via HEPEDOCK. The surface of the receptors and SP40 peptides are shown in wheat and blue colors, respectively. (**A**) Nucleolin, (**B**) Human cyclophilin, (**C**) Human galectin, (**D**) Fibronectin, (**E**) SCARB2, (**F**) DC-SIGN, (**G**) Annexin A2, (**H**) Human tryptophanyl-tRNA synthetase l, (**I**) Vimentin and (**J**) Human prohibitin.

Based on the first round of global molecular docking, the overall binding sites of SP40 peptide are presented in Figure 2 and the top five regions with the highest scores were selected (Supplementary Figure S1) and used for the refinement and local molecular docking. 

Nucleolin is an abundant nucleolar phosphoprotein that can be found in organisms from yeasts to plants and mammals. Nucleolin is located mostly in dense fibrillar regions of the nucleolus and it is also expressed at the cell surface where it acts as a receptor for viruses, such as EV-A71 [21], poliovirus [47] and coxsackieviruses B serotypes 1 to 6 [48]. Nucleolin comprised three structural domains including an acidic N-terminal domain, two to four RNA binding domains (RBD), and a C-terminal RGG rich ‘‘tail’’ which are able to interact with different proteins. [49]. Su et al. [21] showed that nucleolin could bind to EV71-immunoprecipitated particles. The VP1 of EV-A71 was reported to interact directly with nucleolin and the binding of EV-A71 to RD cells could be inhibited by using an anti-nucleolin antibody. In addition, the knockdown of nucleolin expression in RD cells was shown to significantly reduce EV-A71 infection. On the other hand, the expression of human nucleolin in infected murine NH3T3 cells was able to increase the cytopathic effects caused by EV-A71 infection. Therefore, nucleolin was demonstrated to be an attachment receptor responsible for EV-A71 entry [21]. The best available structure of nucleolin in the PDB database was the solution structure of the RBD1,2 domains from human nucleolin (PDB ID 2KRR) that has two domains. The global docking showed that most of the SP40 peptide poses were bound to the region between RBD 1 and 2 domains as shown in Figure 2A and five of the top-scoring complexes were used for local docking. 

Cyclophilins are involved in several cellular processes, such as transcriptional regulation, immune responses, protein secretion, and mitochondrial function [50]. Cyclophilin A is a highly abundant cytosolic protein that plays a significant role in the proliferation of several viruses [51]. Qing et al. [28] showed that cyclophilin A plays important role in EV-A71 proliferation by using cyclophilin A inhibitors as a bioprobe. Cyclophilin A interacted with the VP1 of EV-A71, modified the conformation of the VP1 H-I loop and worked as an uncoating regulator in the EV-A71 entry step. The H-I loop is located in the VP1 of EV-A71 amino acids 239-GSSKSKYPL-247. The uncoating regulatory ability of cyclophilin A has made it different from other attachment receptors reported previously. The cyclophilin A 3D structure comprises an eight-stranded antiparallel β-sheet barrel capped by α-helices [52]. The human cyclophilin A protein structure with PDB ID 5KUW contains 165 amino acids. Global molecular docking showed that all of the SP40 peptide poses were located at the opposite side of the N- and C-terminal region, close to β-strand 3 (Figure 2B). Therefore, the five top-scoring models generated by HPEPDOCK were used for the local docking study. Galectin-1 is a soluble beta-galactoside binding lectin that is expressed in mammalian tissues, such as the brain and lymphoid tissues [53]. It regulates neuron degeneration and immune responses and plays an important role in viral diseases [54,55]. Using confocal microscopy, Lee et al. [27] showed that galectin-1 could colocalize with EV-A71 in SK-N-SH or RD cells. The co-immunoprecipitation of galectin-1 and EV-A71 from infected cells had shown that anti-galectin-1 antibody could precipitate the EV-A71-galectin-1 complex, which was detected using immunoblotting with anti-EV-A71 antibody and the anti-galectin-1 antibody. This indicated the interaction of galectin-1 with the EV-A71 during replication. EV-A71, which was propagated in SK-N-SH cells with galectin-1 gene knockdown, showed lower infectivity in the cell culture and less pathogenicity in the mice compared to the virus that was propagated in the normal cell. Human galectin-1 could bind to the VP1 and VP3 of EV-A71 through carbohydrate residues and it could be released and interact with another cell surface along with the virus. [27]. The overall human galectin-1 3D structure comprises a two antiparallel β-sheet sandwich that adopts a closing hand shape. The backhand is formed by five strands (F1 to F5 which form the F-sheet), while the palm consists of six strands (S1 to S6 that form the S-sheet). The carbohydrate-binding site (CBS) is located in a groove in the S-sheet side of the sandwich, and the β-galactoside recognition core motif is mediated by sheets S4, S5, and S6. The β-galactoside recognition core motif has been used for docking analysis for drug discovery [56]. In the global docking study, using HPEPDOCK, the majority of the poses were located at the groove in the S-sheet side of the sandwich next to the carbohydrate-binding site, but few were bound to the surface of the F-Sheet side of the antiparallel β-sheets. (Figure 2C). However, the top five poses of SP40 peptide were placed at the carbohydrate-binding site which was used for local docking (Supplementary Figure S1). 

Fibronectin is a high-molecular-weight extracellular matrix glycoprotein with a multidomain structure. The arginine-glycine-aspartic acid (RGD) binding site of fibronectin was shown to bind to integrin α5β1, which is a specific receptor for fibronectin. Cells mediate the fibronectin matrix assembly through integrin binding to the RGD (Arg-Gly-Asp) cell-binding domain. [57]. He et al. [23] indicated that the overexpression of fibronectin enhanced EV71 infection, while the knockdown of the fibronectin could greatly reduce viral yield and diminished viral binding to host cells. A synthetic, Arg-Gly-Asp-Ser (RGDS) peptide with the sequence known as the fibronectin motif could significantly inhibit the replication of EV-A71 in cell cultures and neonatal mice. It was shown that the N-terminal fragment of VP1 of EV-A71 interacted with the D2 domain of fibronectin. The finding demonstrated that EV-A71 particles bound to the fibronectin and facilitated virus entry [23]. The only protein structure of the cell-binding domain comprising amino acids 1358–1631 in the protein database which corresponded with the human fibronectin encompassing the RGD loop and synergy region with PDB ID 1FNF. The structure of the fibronectin cell-binding domain contained four contiguous fibronectin-III modules in an extended rod-like molecule with a long axis of around 140 Å. The global molecular docking study showed that most SP40 poses were at the first and third contiguous regions, which also bound to the C-terminal of the protein and close to the RGD loop (Figure 2D). The top 5 scored poses of SP40 peptide were placed at the third contiguous region and C-terminal close to the RGD loop. This region was used for the local docking.

Scavenger receptor class B, member 2 (SCARB2) is a type III transmembrane protein which is known as lysosomal integral membrane protein II and is a major receptor involved in EV-A71 infection. SCARB2 supports the attachment and internalization of EV-A71 and was shown to initiate conformational changes that steer the uncoating of viral RNA in the cytoplasm in RD and Vero cells [58]. The three-dimensional structure of the EV-A71-SCARB2 receptor complex was determined by cryo-electron microscopy [59]. The SCARB2 structure contains a large anti-parallel β-barrel with many short α-helical segments. At the top of the anti-parallel β-barrel, three α-helices, α-4, α-5 and α-7 with another 2 short helices and the β7 strand constitute the head region of the SCARB2 that is considered as the extracellular domain and it is exposed at the cell surface. SCARB2 also has a short cytoplasmic domain at the amino- and carboxy-termini. The structural conformation of SCARB2 is pH-dependent and His-150 plays an important role in switching the neural form that binds to the β-glucocerebrosidase (β-GC) to the acidic form that does not bind. Two α-helices in the head region [α-5 (amino acid no. 153–163) and α-7 (amino acid no. 183–193)] of SCARB2 bind to the GH loop of VP1 and the EF loop of VP2 capsid proteins of EV-A71. SCARB2 has been shown to uncoat the EV-A71 RNA in a low pH-dependent manner [60]. Besides SCARB2, other cell receptors were reported to support the surface binding of EV-A71 [30]. Global docking showed that the majority of the SP40 peptide poses were placed close to the cavity in the large anti-parallel β-barrel and also in the concavity under the head domain of SCARB2 (Figure 2E). The top five scoring poses were placed in the cavity under the head domain, which was used for local docking. 

Dendritic cell-specific intercellular adhesion molecule-3-grabbing non-integrin (DC-SIGN) is a calcium-dependent C-type lectin receptor [61,62] that is expressed in dendritic cells (DCs), macrophages, activated B cells, skin dermis, placenta, intestinal and genital mucosa, and lymphoid tissues [61,63,64,65,66]. It binds to oligosaccharide ligands found on human tissues as well as to pathogens including viruses, bacteria, and parasites. The extracellular portion of this receptor contains a membrane-distal carbohydrate-recognition domain (CRD) and forms tetramers stabilized by an extended neck region consisting of 23 amino acid repeats. The intact receptor has a tetrameric state. Hydrodynamic studies on truncated receptors demonstrate that the portion of the neck of the protein adjacent to the CRD is sufficient to mediate the formation of dimers, whereas regions near the N-terminus are needed to stabilize the tetramers. Crystallization study of truncated receptors showed that CRDs are flexibly linked to the neck, which contains α-helical segments interspersed with non-helical regions. The flexibility of the CRDs in the tetramer along with data on the specificity of these receptors showed a significant role for oligomerization in the recognition of endogenous glycans, in particular those present on the surfaces of enveloped viruses recognized by these proteins [67]. There are a few viruses that can abduct DC-SIGN receptor to spread such as the measles virus, hepatitis C virus and HIV [68,69,70]. Ren et al. [22] reported that monocyte-derived dendritic cells (MDDCs) could capture EV-A71 through viral binding to DC-SIGN, and these surface-bound viral particles could be transferred to susceptible cells for robust infection. The full-length DC-SIGN consists of 268 amino acids encoded by the gene CD209. The 3D structures of DC-SIGN available in the protein database contain 139 to 170 amino acids and the neck region of the structure did not appear in those crystal structures. Therefore, by using five templates of the DC-SIGN retrieved from PDB, a new structure was predicted which contained amino acids 38 to 260. The templates were crystal structures of DC-SIGN_CRD with PDB ID 1SL4 which consists of 155 amino acids, PDB ID 1SL5 which consists of 139 amino acids, PDB ID 2IT5, which consists of 139 amino acids, PDB ID 2IT6 which consists of 155 amino acids and PDB ID 2XR6 which consists of 170 amino acids. The predicted structure of DC-SIGN was used for the docking study. The global docking investigation showed that most of the SP40 peptide poses were bound to the CRD and neck domains on both sides of the protein (Figure 2F). For local docking, the five top-scoring SP40 peptide poses were used. 

Annexin A2 is a calcium- and phospholipid-binding protein that acts as a profibrinolytic co-receptor in tissue plasminogen activator and plasminogen on endothelial cells [71]. Besides that, it is involved in endocytosis, exocytosis, membrane domain organization, actin remodeling, signal transduction, protein assembly, transcription and mRNA transport, and DNA replication and repair [72]. Yang et al. [20] showed that EV-A71 could bind to annexin A2 through VP1 in RD cells. Both pretreatments of EV-A71 with soluble recombinant annexin A2 or pretreatment of host cells with an anti-annexin A2 antibody reduced viral attachment to the cell surface and subsequently reduced the virus yield in vitro. The VP1 amino acids 40 to 100 are the annexin A2-interacting domain that had been mapped by yeast two-hybrid analysis. Annexin A2 was shown in pull-down assay that it could directly bind to five strains of enterovirus 71 belonging to different sub-genotypes C2, C5, and B5 whereas CV-A16 did not bind to annexin A2. Annexin A2 in human cells is encoded by the ANXA2 gene and it is translated to 338 amino acids. The 3D structure of annexin A2 is divided into four homologous repeats (I–IV) and each comprises a structurally similar domain containing five α-helices. The entire molecular structure looks like a curved disk with the four subdomains arranged in a near-parallel pattern. In this study, the crystal structure of a non-mutated and uncomplexed human annexin A2 (PDB ID 2HYW) was used, and it has 30 amino acids in the N-terminal region, which were not visible in the structure [73]. In global docking, most of the SP40 peptide poses were placed between the groove of domains I and II, domains II and III, and domains III and IV, as shown in Figure 2G. The first five top-scoring poses that were used for local docking were located in the groove between domains I and II and domains III and IV. 

Human tryptophanyl-tRNA synthetases (hTrpRS) are enzymes in the human body that exist in two different cytoplasmic and mitochondrial forms. Cytoplasmic hTrpRS catalyzes the aminoacylation of tRNA(trp) with tryptophan and is induced by interferons which are signaling proteins made and released by host cells in response to the presence of several viruses [74]. The 3D structure of hTrpRS comprises three domains: a N-terminal domain up to residue 150, a Rossmann fold (RF) catalytic domain from residues 151–362 and 453–471 and a C-terminal domain from residues 363–452. The catalytic active site is located in a deep pocket of the RF domain and is surrounded by several conserved structural elements, including the KMSAS loop (the connecting loop between strand β9 and helix α16), the HVGH motif (helix α7) and the AIDQ motif (helix α15). The substrate-binding pocket comprises the Trp-binding site and the ATP-binding site. The anticodon-binding site is placed at the tip of the C-terminal domain (residues 479–495) [75]. Yeung et al. [24] discovered hTrpRS as an IFN-γ-inducible EV-A71 cellular entry factor by using genome-wide RNAi library screening. The role of hTrpRS in facilitating EV-A71 entry and infectivity was confirmed by virus attachment, in vitro pulldown, antibody/antigen blocking, and CRISPR/Cas9-mediated deletion. RD cells with the hTrpRS knockdown could be protected to induce CPE in the presence of EV-A71 and reduced viral replication compared to the control wild-type RD cells. The 3D structure of hTrpRS with the PDB ID 2QUJ used in this study contained 471 amino acids. The 87 residues of the N-terminal region of the protein is invisible in the structure. The global docking showed that majority of the SP40 peptide poses were placed close to the catalytic domain, which is responsible for the aminoacylation of tRNA(trp) with tryptophan (Figure 2H). However, the top-scoring SP40 peptide pose was located at the surface between the N and C terminals and catalytic domains. There is doubt that the catalytic domain is involved in EV-A71 attachment or entry of the virus to the cell. However, in local docking, all the five top-scoring poses of HPEPDOCK were used for further evaluations. 

The cell cytoskeletons are made up of microfilaments, microtubules and intermediate filaments (IF). Vimentin is a type III intermediate filament (IF) protein [76] that maintains the cell shape, is responsible for the integrity of the cytoplasm and stabilizes the cytoskeletal interactions. Vimentin is expressed in leukocytes, fibroblasts and endothelial cells of blood vessels. It is also expressed on the cell surface which plays a role in the attachment of several pathogens [77,78,79,80]. Du et al. [25] reported that vimentin could facilitate EV-A71 attachment to the cell. Pull-down assay proved that there was a direct binding of VP1 of EV-A71 to the vimentin receptor. Besides, using soluble vimentin, anti-vimentin antibody, and knockdown of vimentin expression by RNA interference (RNAi) also showed a reduction in EV-A71 infection. A combined treatment of U251 cells with anti-vimentin antibody and anti-SCARB2 antibody was found to be more effective in inhibiting EV-A71 infection. Pull-down assay involving VP1 and truncated fragments of vimentin showed that N-terminal amino acids 1 to 56 of vimentin contained the domain that was directly responsible for the specific binding to VP1. Vimentin self-assembles into a dimer that later forms high-order structures, including tetramers and octamers. However, the details of intermediate filament assembly at crystallographic resolutions are limited to the tetrameric form. The crystal structure of vimentin that was used in this study has been deposited in the protein data bank with PDB ID 5WHF, which is a fragment of a vimentin rod-shaped domain (coil 1B) with a dimer of tetramers in the asymmetric unit. Coil 1B in the crystal is in an infinitely high-order filamentous assembly state, in which the tetramers are packed against each other laterally in an antiparallel fashion across the crystal lattice [81]. Analysis of the global docking of SP40 peptide with vimentin showed that most of the SP40 peptide poses were covering the coil 1B in the middle and the N-terminal areas (Figure 2I). Most of the five top-scoring poses were close to the N-terminal part and was placed on the curved side of the coil 1B, which all used for the local docking study.

Human prohibitin 2 (PHB2) contains 299 amino acids and it is expressed in cell compartments, such as mitochondria, nucleus and plasma membrane. Mitochondria and nucleus prohibitin modulate a variety of signaling pathways controlling cell survival, metabolism and inflammation [82]. The interaction of prohibitin 2 (PHB2) with the C-terminal region of VP1 of EV-A71 was reported to induce autophagy. PHB2 knockdown led to a reduction in EV-A71 replication, viral particle release, viral protein synthesis and the inhibition of autophagy [83]. The available 3D structure of PHB2 in the protein database is the heptad repeat (HR) region of the protein from amino acids 188 to 265 with PDB ID 6IQE that comprises a dimeric, anti-parallel coil–coil [84]. The global docking study showed that most of the SP40 peptide poses were located in the middle of the protein close to the N-terminal region of the 3D structure (Figure 2J). However, the first five top-scoring poses were very close to the N-terminal region of the protein that was used for local docking.

PSGL-1 is a sialomucin leukocyte membrane protein, containing 428 amino acids that functions as a high-affinity counter-receptor for the cell adhesion molecules P-, E- and L-selectins [85,86,87]. PSGL-1 is expressed by lymph node dendritic cells and macrophages in the intestinal mucosa [86]. It plays a significant role in trafficking the leukocytes during inflammation by tethering them to the sites of acute inflammation. The sulfation of the three tyrosine residues at positions 46, 48 and 51, near the N-terminal of PSGL-1, are important for EV-A71 binding. However, PSGL-1 functions as a receptor for only certain EV-A71 strains. These criteria are dependent on the presence of the amino acid 145 in VP1 of EV-A71, which is either glycine or glutamic acid and defines PSGL-1 binding or non-binding to the EV-A71 strains. Sequence analysis of EV-A71 showed that around 80% of strains are not binding to PSGL-1. The cytopathic effects mediated by PSGL-1 binding to EV-A71 could take a longer time (a few days) to be observed in the infected cells compared to EV-A71 that used the SCRB2 receptor. So far, there is no 3D protein structure of PSGL-1 that could be used for molecular docking. PSI blast showed that there is no close structure to the PSGL-1 amino acid sequence for PSGL-1structure prediction. Therefore, PSGL-1 was not included in the molecular docking analysis. 

Heparan sulfate (HS) is a polydisperse linear polymer comprising alternate repeating disaccharide units of N-acetylated or N-sulfated glucosamine and glucuronic acid or iduronic acid joined by (1–4) glycosidic linkage [88] that are highly negatively charged due to the sulfate groups. Tan et al. [19] showed that inhibiting the interaction between HS and EV-A71 by the preincubation of the virus with heparin or protecting the RD cells by preincubating the cells with poly-D-lysine could significantly abolish EV-A71 infection. Treating the cells with heparinase I, II, and III could reduce EV-A71 binding to the surface of RD cells. In addition, blocking the biosynthesis of HS by sodium chlorate or knockdown of N-deacetylases/N-sulfotransferase-1 and exostosin-1 could reduce EV-A71 infection. However, HS was not expressed by EV-A71 infected cells that had high levels of SCARB2 expression, indicating that HS did not participate in the replication or dissemination of the virus in vivo [30]. Since HS is a polydisperse linear polymer and cannot be analyzed by HPEPDOCK, HS was not included in the molecular docking analysis. 

Prior to local docking, five top-scoring models generated from HPEPDOCK for each of the receptors were uploaded to the FelxPepDock server for the high-resolution refinement of the protein–peptide complexes. Several studies showed that FelxPepDock enables one to obtain the sub-Angstrom quality of the protein–peptide structure [89,90]. The top-scoring generated models were energy minimized and used for local docking. 

### 2.2. Local Docking to Identify the Interaction Restraints of Cellular Receptors 

**Figure 3:**
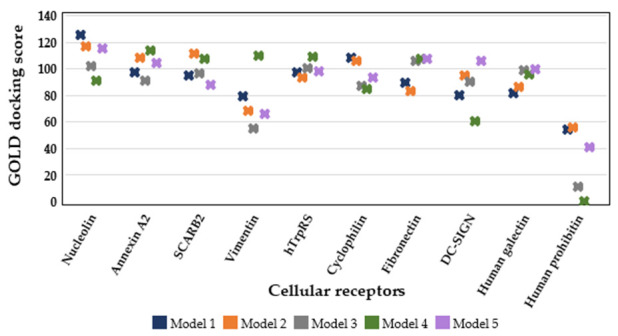
Docking scores (Goldscore) of five top models of receptor-SP40 peptide complexes for each receptor generated by GOLD analysis.

The process of identifying which receptor(s) are most likely to interact favorably with the SP40 peptide is scored using GOLD. Based on the global docking results, five top-scoring complexes were selected to study the interaction of each receptor with the SP40 peptide. For each receptor-SP40 peptide complex, 150 models were generated that were ranked based on GoldScore fitness. The generated results by GOLD for the best score of five top models were presented in Figure 3. In GOLD analysis, the higher fitness represented the higher affinity of the ligand to bind to the receptor. Based on the result, nucleolin demonstrated highest fitness score (125.3) followed by annexin A2 (113.5) and SCARB2(110.9). Only one SP40 peptide complex model with vimentin showed a high score (109.2) and the rest were of lower scores (55 to 79.2) while the five clustered models of human tryptophanyl-tRNA synthetase (hTrpRS) presented scores ranging from 93.0 to 108.5. The reset of receptors showed scattered scores which could indicate the lack of ability to bind to the SP40 peptide. Therefore, the docked complex of SP40 peptide and nucleolin, annexin A2, SCARB2 and hTrpRS were analyzed in detail in this study by Discovery studio software. 

**Figure 4:**
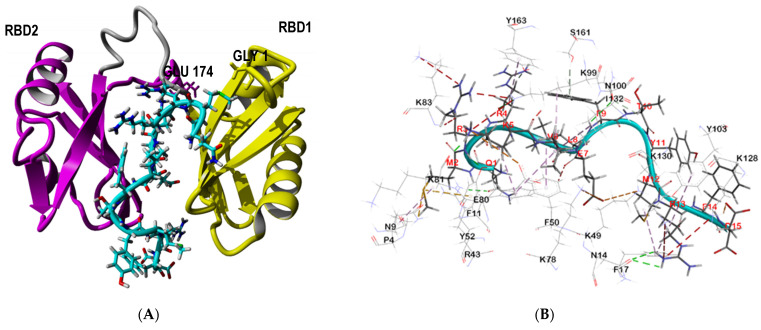
Analysis of interactions between the SP40 peptide with nucleolin. (**A**) The binding pose of SP40 peptide on nucleolin receptor, with SP40 peptide being placed between the RNA binding (RBD) domains of nucleolin. (**B**) Residues in nucleolin involved in interactions with the SP40 peptide. Residues in the target receptors are shown as lines, while residues in the SP40 peptide are shown in sticks. Salt bridge and electrostatic interactions are in orange color, the conventional hydrogen bonds are shown in green dash line, carbon hydrogen bonds are in light green color, sulfur and hydrophobic interactions are in dark yellow and pink colors, respectively.

Nucleolin displayed higher fitness score when the SP40 peptide was docked to the area between its two RNA binding domains (RBDs). The binding pose of the SP40 peptide on the nucleolin receptor is shown in Figure 4A, while its interactions with the amino acid residues of the receptor are shown in Figure 4B. Table 1 shows the residues involved and the type of interactions between nucleolin and SP40 peptide. In the docked complex, the two RBDs and the linker interacted extensively with the SP40 peptide, 11 amino acids of RBD1 (Pro4, Asn9, Phe11, Asn14, Phe17, Arg43Lys49, Phe50, Tyr52, Lys78 and Glu80), 7 amino acids of RBD2 (Lys99, Asn100, Tyr103, Lys128, Ile132, Ser161 and, Tyr163) and 1 amino acid of the linker (Lys81) mediated interactions with the SP40 peptide. In total, there were 1 salt bridge, 3 electrostatic interactions, 11 hydrogen bonds, 2 sulfur interactions and 10 hydrophobic interactions found between nucleolin and the SP40 peptide. Among the 15 amino acids of SP40 peptide, 2 amino acids acted as hydrogen bond donors (Arg3, and Thr10), 5 amino acids acted as hydrogen bond acceptors (Gln1, Glu7, Leu8, Met12 and Asp15) and 6 amino acids were both hydrogen bond donors and acceptors (Met2, Arg4, Lys5, Val6, Phe9 and Arg13). Amino acids Tyr11 and Phe14 of SP40 peptide were not involved in interactions with nucleolin. 

Alanine scanning of the SP40 peptide showed that replacing the amino acids Arg3, Arg4, Lys5, Met12 and Arg13 with Ala residue could decrease 60 to 80% inhibitory effects of the SP40 peptide [31]. Analyzing the docked complex of SP40 peptide with nucleolin showed that Arg3 is interacting with Tyr52 to form a hydrogen bond and Arg 4 is interacting with Lys99 and Tyr163 to form two hydrogen bonds and with Tyr163 to form one hydrophobic interaction. Lys5 is involved in 3 electrostatic, hydrogen bond and hydrophobic interactions with Glu80, Lys81 and Phe11, respectively. Aside from this, Met12 formed one hydrophobic interaction with Phe17 while Arg13 formed three hydrogen bonds and two hydrophobic interactions.

Tan et al. [31] showed that the SP40 peptide does not bind to EV-A71 and it is not virucidal to EV-71 viral particles [31]. Since pre-incubation of the RD cell with the SP40 peptide prior to EV-A71 infection showed around 90% inhibitory effects on plaque formation and RNA copy number reduction in EV-A71 when compared to the addition of SP40 peptide during post-infection where very minor inhibition of viral infectivity was observed (Unpublished observation), indicating that SP40 peptide was interacting with cell receptor and preventing EV-A71 at the attachment or entry-stage. However, Su et al. [21] showed that the knockdown of cell surface nucleolin significantly decreased EV-A71 infection and RNA production in human cells. The EV-A71 RNA levels were decreased by 25% and 45% in the nucleolin-knockdown cells at 3 and 6 h post-infection, respectively. 

The SP40 peptide had demonstrated broad antiviral activities against EV-A71, CV-A16 and poliovirus type 1 in RD cell cultures [31]. Meanwhile, Waggoner and Sarnow (1998) showed nucleocytoplasmic relocalization of nucleolin into the cytoplasm following poliovirus infection [91]. Izumi et al. [47] showed that nucleolin stimulated viral IRES-mediated translation both in vitro and in vivo. A nucleolin mutant lacking the RBD1 could not perform IRES-mediated translation in vitro, which highlighted the role of RBD1 in viral protein translation [47]. Since poliovirus and EV-A71 both use viral IRES-mediated translation, nucleolin might have the same function in the translation of EV-A71. Hence, blocking nucleolin using the SP40 peptide might inhibit the translation of EV-A71. As the entire structure of nucleolin is not available in the protein database, it is not possible to predict the interaction of SP40 peptide with the acidic N-terminal domain or the C-terminal RGG rich tail of nucleolin which could be involved in the attachment of EV-A71 to nucleolin. 

**Figure 5:**
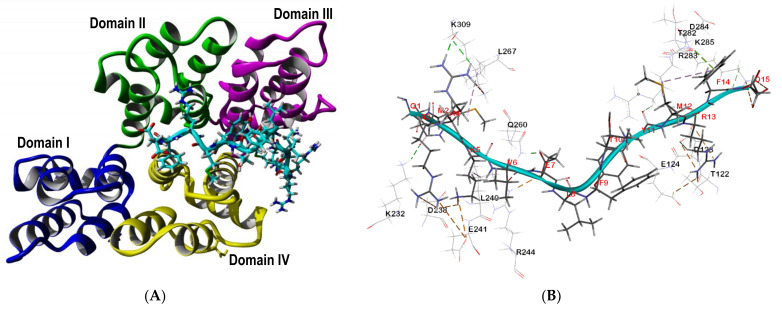
Analysis of interactions between SP40 peptide with annexin A2. (**A**) The binding pose of SP40 peptide on annexin A2 receptor. (**B**) Residues in annexin A2 involved in interactions with the SP40 peptide. Residues in the target receptors are shown as lines, while residues in the SP40 peptide are shown in sticks. Salt bridge and electrostatic interactions are in orange color, the conventional hydrogen bonds are shown in green dash line, carbon–hydrogen bonds are in light green color and hydrophobic interactions are in pink color.

Annexin A2 in local docking analysis with the SP40 peptide as a ligand showed the second-best fitness GoldScore compare to the other cellular receptors. The complexes of the SP40 peptide compared to different regions of annexin 2 in 5 different models were studied. The best Gold-score belongs to the SP40 peptide docked in the cavity area between domains II, III and IV on the opposite side of the N- and C-terminals regions (Figure 5A). The SP40 peptide interactions with the amino acid residues of the receptor are shown in Figure 5B. Post docking analysis of SP40 peptide-annexin A2 complex revealed 26 interactions comprising 4 salt bridges, 6 electrostatic and 11 hydrogen bonds, 1Pi-lone pair and 4 hydrophobic interactions between the SP40 peptide and annexin A2 receptor (Table 2). In the docked complex, three domains of annexin A2 vastly interacted with the SP40 peptide, 3 amino acids of domain II (Ther122, Asp123 and Glu124), 6 amino acids of domain III (Lys232, Asp238, Leu240, Glu241, Arg244, and Gln260) and 7 amino acids of domain IV (Leu267, Thr282, Arg283, Asp284, Lys285 and Lys309) mediated interactions with the SP40 peptide. Among 15 amino acids of the SP40 peptide, 3 amino acids acted as hydrogen bond donors (Arg3, Arg4 and Lys5,), 6 amino acids acted as hydrogen bond acceptors (Gln1, Met2, Glu7, Tyr11, Met12 and Asp15), 2 amino acids were both hydrogen bond donors and acceptors (Arg13 and Phe14) and 4 amino acids showed no interactions (Val6,Leu8, Phe9, and Thr10). Arg 3 formed two electrostatic interactions and Arg4 formed two hydrogen bonds with amino acids in domains III and IV, respectively. Lys5, and Asp15 also mediated two salt bridges, two electrostatic interactions with amino acids in domain III and IV. Glu7 formed one salt bridge and one hydrogen bond with amino acids in domain III while Arg13 made one salt bridge, five hydrogen bonds and two electrostatic interactions with amino acids in domain II and III.

The overall annexin molecule is planar and curved with opposing concave and convex surfaces. The concave face of the protein faces the cytosol and contains both the N-terminal and C-terminal domains. The convex surface lies along the plane of the phospholipid membrane and also contains the interhelical loops and seven Ca^2+^-binding sites. The common heparin-binding site is situated at the convex face of domain IV of annexin A2. At this site, annexin A2 binds up to five sugar residues from the nonreducing end of the oligosaccharide. One of these Ca^2+^-binding sites lies between the IV-AB (Gly279–Arg283) and IV-DE (Thr322–Tyr326, including the N-terminal end of helix E of domain IV) loops in domain IV of annexin A2, where it participates in heparin binding. For heparin-binding at this site, main-chain and side-chain nitrogen atoms and two calcium ions play important roles in the binding [73]. The IV-AB loop conformation stabilization is through the coordination of binding to the Ca^2+^ ion and it provides a pre-formed binding surface suitable for oligosaccharide residue. In addition to the protein-bound Ca^2+^ ion, heparin interacts with annexin A2 through polar interactions, mostly between its sulfated oxygen atoms and the nitrogen atoms from the IV-AB and IV-DE loops residues of annexin A2. Interestingly, SP40 peptide binds to the residues in the IV-AB loop (Thr282 and Arg283) of domain IV by two hydrogen bonds, two hydrophobic and one Pi-lone pair interactions which could alter the conformation of the IV-AB and disrupt the Ca^2+^ binding and subsequently prevent the heparin-binding.

Furthermore, the nonreducing end of the oligosaccharide binds at the IV-AB loop, consistent with a possible physiological recognition site for the nonreducing end of the free heparin polymer or the heparan sulfate (HS) glycosaminoglycan chain attached to a proteoglycan [73]. The binding of SP40 peptide to domain IV of annexin might prevent the covalent attachment of HS to core proteins such as annexin A2, which could inhibit the attachment or entry of the EV-A71 to the target cell through HS.

**Figure 6:**
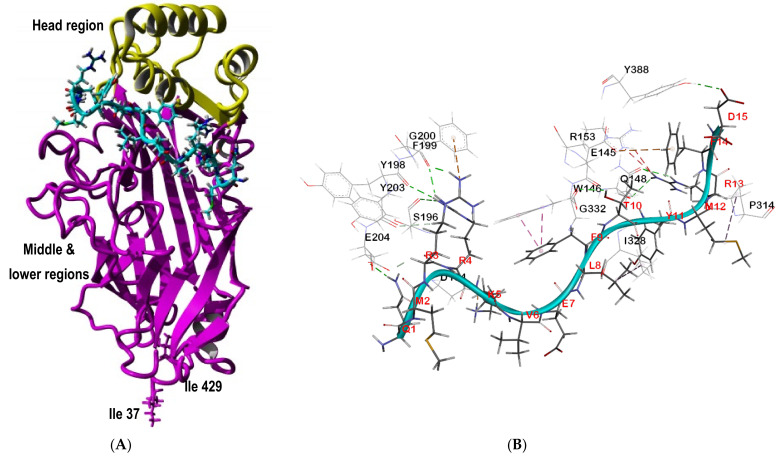
Analysis of interactions between SP40 peptide with SCARB2. (**A**) The binding pose of SP40 peptide on SCARB2 receptor. (**B**) Residues in SCARB2 involved in interactions with the SP40 peptide. Residues in the target receptors are shown as lines, while residues in the SP40 peptide are shown in sticks. Salt bridge is in orange color, the conventional hydrogen bonds are shown in green dash line, carbon hydrogen bonds are in light green color, electrostatic interaction is in yellow and hydrophobic interaction is in purple color.

Local docking analysis of the SP40 peptide under the head region of SCARB2 showed a better fitness score compared to the docking of the peptide to the large anti-parallel β-barrel in the middle domain (Model 5, Figure 3). SCARB2 was the third receptor that showed a high score to bind to the SP40 peptide. Local docking analysis of the concavity under the head domain of SCARB2 exhibited a higher score compared to the pose that SP40 peptide extensively binds to the head domain (Model 3, Figure 3). The binding pose of the SP40 peptide on the SCARB2 receptor is shown in Figure 6A and SP40 peptide interactions with the amino acid residues of the receptor are presented in Figure 6B. Analysis of the docked complex of SP40 peptide-SCARB2 showed that six amino acids of the head region (Glu145, Trp146, Gln148, Asp194, Ser196 and Tyr198) and eight amino acids of the middle region (Phe199, Gly200, Tyr203, Glu204, Pro314, Ile328, Gly332 and Tyr388) of SCARB2 interacted with SP40 peptide (Table 3). 

Post docking analysis of the complex of SP40 peptide-SCARB2 revealed 19 interactions, 1 salt bridge, 12 hydrogen bonds, 2 electrostatic Pi- interactions and 4 hydrophobic interactions between the SP40 peptide and SCARB2 receptor. Among 15 amino acids of SP40 peptide, six amino acids acted as hydrogen bond donors (Gln1, Arg3, Arg4, Lys5, Thr10and Arg13) and five amino acids acted as hydrogen bond acceptors (Leu8, Phe9, Met12, Phe14 and Asp15). Furthermore, four amino acids of SP40 peptide (Met2, Val6, Glu7 and Tyr11) did not form any interaction with SCARB2. Arg3 of SP40 peptide formed four hydrogen bonds and an electrostatic- Pi-Cation interaction while Arg4 formed two hydrogen bonds. Lys5 mediated a salt bridge and Gln1 and Arg 13 were involved in one and three hydrogen bonds with SCARB2, respectively. Asp15 as an acceptor residue formed one hydrogen bond while Leu8, Phe 9 and Met12 were involved in hydrophobic interactions and Phe 14 formed an electrostatic -Pi anion interaction. 

In the 3D structure of SCARB2, α-helix 4 (amino acids 138–146) and α-helix 5 (amino acids 149–163) are linked by Ser147 and Gln148. The α-helix 7 (amino acids 182–189) is linked to the middle domain by amino acids 190–198. These helices were reported to bind to the GH loop of VP1 and the EF loop of VP2 capsid proteins of EV-A71 [60]. Interestingly, the analysis of the SP40 peptide-SCARB2 complex displayed that Glu145 and Trp146of α-helix 4 acted as a hydrogen donor residue and formed one electrostatic and two hydrophobic interactions with Phe14 and Phe9 of SP40 peptide. Additionally, three amino acids (Asp194, Ser196 and Tyr198) that linked the head domain helices to the middle domain formed one salt bridge and five hydrogen bonds with SP40 peptide. Binding of SP40 peptide to the α-helix 4 and linker of the α-helix 7 might inhibit the binding of EV-A71 to SCARB2 and prevent virus entry into the cells. The interaction of the SP40 peptide to the head and middle regions of the SCARB2 might change the structural conformation of the receptor. This structural alteration of the receptor might prevent the attachment of the receptor to the capsid VP1 protein of EV-A71 and subsequently, reduced the infection rate of the virus. 

**Figure 7:**
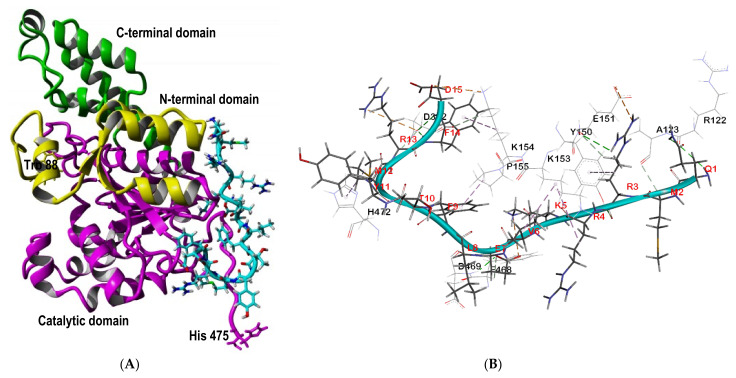
Analysis of interactions between SP40 peptide with human tryptophanyl-tRNA synthetase (hTrpRS). (**A**) The binding pose of SP40 peptide on hTrpRS receptor. (**B**) Residues in hTrpRS involved in interactions with the SP40 peptide. Residues in the target receptors are shown as lines, while residues in the SP40 peptide are shown in sticks. Electrostatic interactions are in orange color, the conventional hydrogen bonds are shown in green dash line, carbon hydrogen bonds are in light green color and hydrophobic interactions are in pink color.

The local molecular docking of five top-scoring complexes of human tryptophanyl-tRNA synthetases (hTrpRS) with SP40 peptide had revealed the fourth highest fitness score among the other analyzed cellular receptors (Figure 3) when the SP40 peptide was docked to the cavity between N-terminal and C-terminal domains and between N-terminal and the catalytic domain of hTrpRS (Supplementary Figure S1A). The binding pose of the SP40 peptide on the hTrpRS receptor is shown in Figure 7A, while its interactions with the amino acid residues of the receptor are presented in Figure 7B. The residues involved and the type of interactions between hTrpRS and SP40 peptide are shown in Table 4. In the docked complex, the hTrpRS interacted with the SP40 peptide with the formation of 12 hydrogen bonds, 3 electrostatic, 1 Pi-cation and 6 hydrophobic interactions. The amino acids of hTrpRS that mediated the interaction with SP40 peptide belonged to the N-terminal domain (Arg122, Ala123 and Tyr150) and catalytic domain (Glu151, Lys153, Lys154, Pro155, Phe468, Asp469 and His472). 

Moreover, six amino acids of SP40 peptide are interacting with hTrpRS, and they acted as hydrogen bond donors (Gln1, Met2, Arg4, Lys5, Phe9 and Arg13), while Val6, Glu7 and Met12acted as hydrogen bond acceptors and Arg3, Phe14 and Asp15 were both hydrogen bond donors and acceptors. Arg3 formed three hydrogen bonds with Tyr150 and Glu151 and one electrostatic and one hydrophobic interaction with Glu151 and Tyr150, respectively. Arg122 and Ala123 of the N-terminal domain of hTrpRS mediated three hydrogen bonds with Gln1 and Met2 of SP40 peptide. Glu7, Arg13 Phe14, Asp15 formed six hydrogen bonds with amino acids in the catalytic domain of hTrpRS. 

The hTrpRS is involved in aminoacylation reaction in which the tRNA acceptor arm interacted with helix α1′ (amino acids 100–108) of the N-terminal domain and helices α6 (amino acids 259–265) and α9 (amino acids 321–329) of the catalytic domain. Helix α1′ acted as a platform at the entrance to the catalytic active site to hold the tRNA 3′ end. The 3′ end anticodon took a sharp turn to enter into the catalytic active site with a deformed conformation. The nucleotide base interacted with three residues (Asp99, Lys102 and Arg106) of helix α1′ [92]. Conformational changes caused by binding to the SP40 peptide might affect the aminoacylation reaction.

As there is no mechanism of action study related to the attachment or entry of EV-A71 through the hTrpRS receptor, it would be difficult to justify the docking result. However, if EV-A71 used the tRNA acceptor arm to interact with the N-terminal for attachment or entry, it would be affected by the presence of SP40 peptide which could bind to Arg122 and Ala123 of helix α2′ and change the conformation of the hTrpRS receptor. 

The finding of the molecular inhibition mechanism of the SP40 peptide was further verified through receptor-blocking experiments. Seven receptors (nucleolin, annexin A2, PSGL-1, vimentin, heparan sulfate, galectin-1andSCARB2) of EV-A71 expressed in RD cells were blocked by the respective anti-receptor antibodies and their inhibitory effects in the presence and absence of SP40 peptide were analyzed.

### 2.3. SP40 Peptide Worked Synergistically with Anti-Receptor Antibodies

Attachment or entry of EV-A71 to seven receptors of EV-A71 were selectively blocked with their respective antibodies followed by L-SP40 peptide (100 μM) treatment. The results indicated that the L-SP40 peptide synergistically enhanced the inhibitory effects of antibodies to specific receptors when they were blocked with their respective antibodies and subsequently incubated with the L-SP40 peptide. All the antibodies were used at the maximum concentration which was found to be non-cytotoxic. With the exception of annexin A2 at 10 µg/mL, all the other antibodies were used at 20 µg/mL. 

**Figure 8:**
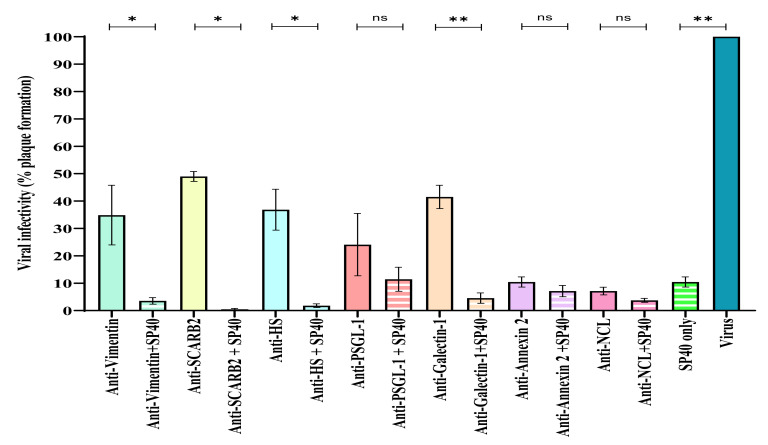
Effects of L-SP40 peptide with specific anti-receptor antibodies on viral infectivity. RD cells were treated with antibodies targeting various receptors for 1 h followed by incubation with 100 µM L-SP40 peptide for 1 h. The L-SP40 peptide-treated cells were subsequently infected with EV-A71 (MOI = 0.1) for 1 h. Viral infectivity was determined by plaque assays. Error bars indicate the range of values obtained from three independent experiments (* *p* < 0.05, ** *p* < 0.01, ns = non-significant analyzed by *t*-test).

Anti-nucleolin and anti-annexin A2 receptors when blocked with their respective antibodies exhibited the highest inhibition of EV-A71 infection at 92.88% and 89.55%, respectively (Table 5). The residual viral infectivity exhibited by the virus was found to be significantly lower than the untreated the antibodies against the other receptors (anti-vimentin, anti-heparan sulfate, anti-PSGL virus control (Figure 8). Treatment with -1 and anti-galectin-1) were observed not to exhibit such high levels of viral inhibition when RD cells were treated with them. When SCARB2, a critical uncoating receptor of EV-A71 was blocked with an anti-SCARB2 antibody, the inhibition of viral infectivity was found to be 51%. Similarly, antibodies against attachment receptors, such as heparan sulfate or galectin-1 could only inhibit infectivity by 63.1% and 58.5%, respectively. Other anti-receptor antibodies, such as anti-vimentin and anti-PSGL-1 antibodies, were able to inhibit the infectivity at 65.1% and 75.9%, respectively. The very high levels of inhibition of infectivity by anti-nucleolin and anti-annexin A2 antibodies indicated that these two receptors played very significant roles as receptors for EV-A71 interactions in comparison with the remaining receptors being evaluated.

Interestingly, when L-SP40 peptide was added to RD cells that were blocked by anti-receptor antibodies, there was a significant increase in the inhibition of viral infectivity to more than 90% except for anti-PSGL-1, where the reduction was found to be 88.5%. When anti-annexin A2 and anti-nucleolin blocked cells were treated with L-SP40 peptide, the inhibition increased slightly from 89.5% to 92.87% and from 92.8 % to 96.28%, respectively. The maximum inhibition of infectivity (99.54 %) was observed when L-SP40 peptide was added to the SCARB2 receptor blocked with anti-SCARB2compared to all treatment groups. Blocking the other receptors with their respective antibodies, except for annexin A2, showed that the inhibitions of viral infectivity were lower than that achieved by anti-nucleolin treatment. Therefore, when each of the receptors (except for nucleolin) were treated with their respective antibodies together with SP40 peptide, the unblocked nucleolin receptor was able to interact with SP40 peptide and this inhibition further contributed to the significant decrease in viral infectivity.

However, since anti-nucleolin and anti-annexin A2 antibodies together with SP40 peptide exhibited quite similar inhibition (96.3% versus 92.8%), it is possible to speculate that SP40 peptide could also bind to annexin A2. In cells treated with anti-nucleolin and SP40 peptide, the residual infectivity could be explained by EV-A71 interaction with other receptors that showed a low probability of binding with SP40 peptide such as human galectin and prohibitin (Figure 3). The results indicated that EV-A71 could use more than one receptor to attach and enter the RD cells because none of the receptor-specific antibodies could provide complete inhibition of infectivity of EV-A71. However, blocking the nucleolin and annexin A2 receptor showed minimal residual infectivity at 7.12% and 10.45%, respectively (Figure 8), indicating that these two receptors are more crucial for attachment and entry of EV-A71 strain 41 into the RD cells. Further decrease in residual infectivity was observed with the addition of L-SP40 peptide to the RD cells which were prior blocked with specific anti-receptor antibodies. Since peptide L-SP40 was demonstrated to bind to nucleolin receptor, the above observations suggested that blocking of other receptors, such as SCARB2, heparin sulfate, vimentin and galectin-1 with their respective antibodies, and the L-SP40 peptide simultaneously could be beneficial in achieving augmented inhibitions of EV-A71. 

It is interesting to note that despite blocking the nucleolin receptor, the virus was still able to enter the cells and replicate, albeit at low infectivity of 7.12%. This finding suggested that EV-A71 could use more than one receptor to enter the cells and continue to replicate. The results were in agreement with the previous study by He et al. [23] who suggested that blocking or knockdown any of the receptors would not completely abolish the infection but would only decrease infection [23]. Our observations also indicated that antibody treatments to block specific receptors of EV-A71 did show a reduction in infectivity such as treatment with anti-SCARB2 resulted in 49% residual infectivity but could not completely abolish infectivity. Hence, treatment directed towards a single attachment or entry receptor of EV-A71 with a specific antibody alone would not be a suitable option for receptors other than nucleolin and annexin A2. 

Interestingly, when the RD cells were blocked by anti-receptor antibodies and subsequently treated with L-SP40 peptide, a significant increase in inhibition of infectivity was observed. Remarkably, when the L-SP40 peptide was used in conjunction with the anti-SCARB2 receptor antibody, 99.5% inhibition with less than 0.5% residual infectivity was observed. This observation indicated that blocking the SCARB2 (uncoating receptor) with an anti-SCARB2 antibody followed by treatment with L-SP40 peptide could significantly reduce the viral infectivity to only 0.46%.

## 3. Materials and Methods

### 3.1. Software Used for Molecular Docking

Docking studies were performed using three different software packages: HPEPDOCK (http://huanglab.phys.hust.edu.cn/hpepdock/) (accessed on 5 August 2021) [35], FlexPepDock (http://flexpepdock.furmanlab.cs.huji.ac.il/) (accessed on 15 August 2021) [45], GOLD Suite (Hermes 2020.3.0) [41] and YASARA v21.6.17 software (http://www.yasara.org/) (accessed on 5 August 2021) used to energy minimized the 3D-structure of the receptors and receptor-SP40 peptide complexes. The SP40 peptide structure was modeled using the PEP-FOLD online server (https://bioserv.rpbs.univ-paris-diderot.fr/services/PEP-FOLD/) (accessed 2 March 2021). All the cell receptors and peptide structures were energetically minimized using YASARA software and saved as a PDB file format.

The molecular docking calculation and data analysis were performed on an Intel(R) Xeon(R) W-2135 CPU @ 3.70GHz- based machine running MS Windows 10 as an operating system.

### 3.2. Preparation of Cell Receptors and SP40 Peptide

The protein structures of the following receptors were retrieved from the Protein Data Bank (https://www.rcsb.org/) (accessed on 10 March 2021): human galectin-1 (PDB ID: 1GZW) with a resolution of 1.70 Å, nucleolin (PDB: 2KRR) (solution NMR), SCARB2 (PDB ID: 4F7B) with a resolution of 3.006 Å, fibronectin (PDB ID: 1FNF) with a resolution of 2.00 Å, human prohibitin (PHB) (PDB ID: 6IQE) with a resolution of 1.70 Å, human tryptophanyl-tRNA synthetase (PDB ID: 2QUJ) with a resolution of 2.42 Å, Annexin A2 (PDB ID: 2HYW) with a resolution of 2.10 Å, human cyclophilin (PDB ID: 5KUW) with a resolution of 1.70 Å and vimentin (PDB ID: 5WHF) with a resolution of 2.25 Å. 

The structure of dendritic cell-specific intercellular adhesion molecule-3-grabbing non-integrin (DC-SIGN) was predicted by YASARA software (http://www.yasara.org/) (accessed on 5 August 2021) based on default setting using 5 templates of the DC-SIGN retrieved from the PDB; crystal structures of DC-SIGN_CRD with PDB ID 1SL4 with a resolution of 1.55 Å, PDB ID 1SL5 with a resolution of 1.80 Å, PDB ID 2IT5 with a resolution of 2.40 Å, PDB ID 2IT6 with a resolution of 1.95 Å and PDB ID 2XR6 with a resolution of 1.35 Å. Meanwhile, there is no available structure of PSGL-1 and sialic acid in the database. 

The docking was performed on one chain of all cell receptor structures. The structures were prepared using YASARA by removing the water molecules, ligands and hetatoms. The hydrogen atoms were added to the structure for optimization and it was energy minimized and saved in active pdb format files.

The amino acid sequence of the SP40 peptide (QMRRKVELFTYMRFD) was submitted to the PEP-FOLD online server to model the three-dimensional (3D) structure of the peptide. The predicted model of the SP40 peptide in the pdb format file was subjected to YASARA where hydrogen atoms were added, energy minimized and saved in the active pdb format file. 

#### Docking Procedure

HPEPDOCK. In global docking, the pdb files of receptors and SP40 peptide were uploaded to http://huanglab.phys.hust.edu.cn/hpepdock/ server (accessed on 5 August 2021). Default values of all parameters were used. By default, the number of generated peptide conformations was set to 1000 in the webserver and the top 100 peptide binding models were selected. As no information about the binding site was provided, the sphere points (represent the negative images of the molecular shape generated by SPHGEN), covering the whole receptor, were clustered and used to represent the possible peptide binding positions on the receptor. Then, the putative peptide binding modes were globally sampled by matching the peptide atoms with the sphere points [93]. The modified version of MDock was used to dock the ensemble of peptide conformations against the whole receptors [94,95]. In HPEPDOCK, all of the sampled binding modes were ranked by an iterative knowledge-based scoring function. The sampled binding orientations were optimized by a SIMPLEX minimization algorithm guided by their binding energy scores, in which both the receptor and the peptide were treated as rigid bodies [96]. The final 100 docking models were provided and saved. The conformation poses of the cell receptor and the SP40 peptide were viewed by the PyMOL software.

FlexPepDock. The refinement of the receptor-SP40 peptide complex was performed using FlexPepDock. The set of five top-scoring receptor-SP40 peptide complexes for each receptor from HPEPDOCK was selected, cleaned and energy minimized by YASARA. Then, the pdf file of the receptor-SP40 peptide complex was uploaded to http://flexpepdock.furmanlab.cs.huji.ac.il/ server (accessed on 15 August 2021). The server docked the peptide starting from initial conformation as uploaded in the receptor-peptide complex pdf file. By default, the server was used to perform 100 simulations in full-atom mode and 100 simulations that included a preceding low-resolution centroid-based optimization protocol [89]. Then, it ranked a total of 200 models created by their Rosetta energy score and provided the top 10 predicted models for the interaction, as well as their score and bb-RMSD from the starting conformation.

GOLD. Local docking was performed using the GOLD (Genetic Optimization for Ligand Docking) program. GOLD uses a genetic algorithm that explores the binding pocket and searches for the best ligand interactions [41]. The energy minimized receptor-SP40 peptide complex was loaded to the Hermes visualizer in the GOLD suite. The protein receptor was prepared by adding hydrogens and extracting the SP40 peptide ligand. The receptor-binding site was defined by reloading the extracted SP40 peptide. The search efficiency of the genetic algorithm was set to “automatic” and the option of “early termination” was switched off. The docking poses were ranked by the GoldScore scoring function. For docking with high accuracy, the number of “GA runs” was set to 150 (peptide length ×10). All other parameters were set as default. Then, according to GoldScore, the conformations for each peptide were collected and reranked. Higher GOLD scores represent a greater affinity of the ligand for the protein at the defined binding site.

### 3.3. Analysis of Docking Results

The 10 top-scoring solutions among the 150 runs provided by GOLD were exported using Hermes analyzed and visualized in Discovery Studios 4.2. The list of interactions was recorded and the bonds between the ligand (SP40 peptide) and receptors were analyzed. 

### 3.4. Receptor Blocking

Human Rhabdomyosarcoma cells (RD) were grown overnight in wells within a 96-well plate. Cells were treated with specific antibodies against each of the receptors. Anti-annexin 2 (Novus Biological, Colorado, CO, USA), anti-vimentin (Santa Cruz Biotechnology, Dallas, TX, USA), anti-SCARB-2 (R&D System, Minneapolis, MN, USA), anti-PSGL-1 (Santa Cruz Biotechnology, Dallas, TX, USA), anti-heparan sulfate (Merck, Rahway, NJ, USA), anti-galectin-1 (Abcam, Cambridge, UK) or anti-nucleolin (Abcam, Cambridge, UK) were incubated with RD cells for 1 h at 37 °C to block the receptors. After one hour, cells were washed with PBS and infected with EV-A71 (MOI 0.1) for 1 h. The inoculum was removed and treated cells were infected with EV-A71 (MOI 0.1) for 1 h. The inoculum was removed and DMEM media containing 2% FBS was added. After 24 h of incubation, the supernatants were collected to determine the virus titers by plaque assays as described by Lalani et al. [97]. 

In the next experiment, the RD cells treated with the each of individual anti-receptors were further incubated with SP40 peptide (100 µM) for 1 h at 37 °C. Cells were then washed with PBS and infected with EV-A71 (MOI 0.1) for 1 h. The inoculum was removed and DMEM media containing 2% FBS was added. After 24 h of incubation, the supernatants were collected to determine the virus titers by plaque assays. 

Table 6 lists the details of the antibodies used in the experiment. The exact binding sites for most of the antibodies are proprietary information that were not available. All the antibodies were used at the maximum concentration which were found to be non-cytotoxic.

## 4. Conclusions

Blocking the nucleolin receptor with anti-nucleolin showed the highest inhibition of viral infectivity and supported the local docking data predicted by the GOLD software. The fitness Goldscore of annexin A2, SCARB2 and human tryptophanyl-tRNA synthetase from local docking also indicated that they are likely to be involved in binding to EV-A71. The inhibition of SCARB2 by anti-SCARB2 only showed inhibition of infectivity at 51.04% but upon adding SP40 peptide which blocked nucleolin, the inhibition of infectivity rose significantly to 99.54%. The data indicated that the likelihood of treating EV-A71 infectious with anti-SCARB2 and SP40 peptide

Furthermore, local docking showed the ability of SP40 peptide for binding to different cell receptors such as nucleolin, annexin A2, SCARB2 and hTrpRS with very close fitness scores ranging from 125.3 to 108.5. The binding of SP40 to these receptors could inhibit the function of the receptors and inhibit any viruses that employ these receptors for their infectivity. For example, the binding of SP40 peptide to the RBDs of nucleolin was shown to inhibit poliovirus and hepatitis C virus (HCV) IRES-mediated translation. Das et al. [98] reported the inhibition of poliovirus and HCV in the presence of small (60 nt) RNA (IRNA) from *Saccharomyces cerevisiae* which was later shown by Izumi et al. [47] that the binding of IRNA to the RBDs of nucleolin could inhibit the translation of poliovirus.

Besides nucleolin acting as the receptor for SP40 peptide, other receptors, such as annexin A2 and hTrpRS could serve as an additional attachment receptor. To further elucidate the number of receptors involved in interacting with SP40 peptide to prevent the EV-A71 infection, knockdown of each of the receptors in RD cell could provide further insight.

Thus, interactions analysis of the SP40 peptide to the cell receptors provided a clue for possible broad-spectrum antiviral activity of SP40 peptide against other viruses which use the same receptor(s) for infectivity.

Meanwhile, this study highlighted the lack of high-resolution structures of several receptors. Additionally, the co-crystallization of peptides with any of the receptors in the study could provide more information on the potential binding site of the receptors that can be used for further in silico study to design new therapeutic peptides.

## Figures and Tables

**Table 1:** Residues in nucleolin involved in interactions with the SP40 peptide.

No	H-Donor Residue	Position	H-Acceptor Residue	Position	Distance	Type of Interaction
1	LYS128	HZ2	***ASP15***	OD1	2.13	Salt Bridge
2	LYS49	NZ	***GLU7***	OE2	5.21	Electrostatic
3	LYS78	NZ	***GLU7***	OE2	4.44	Electrostatic
4	***LYS5***	NZ	GLU80	OE1	4.91	Electrostatic
5	ARG43	HH21	***GLN1***	OE1	2.55	Conventional hydrogen bond
6	LYS99	HZ1	***ARG4***	O	2.76	Conventional hydrogen bond
7	***ARG3***	H13	TYR52	OH	1.72	Conventional hydrogen bond
8	***LYS5***	H14	LYS81	O	1.79	Conventional hydrogen bond
9	***PHE9***	H14	ASN100	OD1	3.02	Conventional hydrogen bond
10	***ARG13***	H14	ASN14	OD1	2.65	Conventional hydrogen bond
11	***ARG13***	H14	ASN14	OD1	2.54	Conventional hydrogen bond
12	***ARG4***	H14	TYR163	OH	1.92	Carbon hydrogen bond
13	***THR10***	H13	ASN100	OD1	2.57	Carbon hydrogen bond
14	***ARG13***	H14	TYR103	OH	2.15	Carbon hydrogen bond
15	***MET2***	SD	ASN9	ND2	3.23	Sulfur-X
16	SER161	HG	***PHE9***	Pi-Orbitals	3.22	Pi-Donor Hydrogen Bond
17	***MET2***	SD	TYR52	Pi-Orbitals	4.37	Pi-Sulfur
18	PRO4	Alkyl	***MET2***	Alkyl	4.93	Hydrophobic-Alkyl
19	LYS99	Alkyl	***VAL6***	Alkyl	5.29	Hydrophobic-Alkyl
20	***VAL6***	Alkyl	ILE132	Alkyl	4.79	Hydrophobic-Alkyl
21	PHE11	Pi-Orbitals	***LYS5***	Alkyl	4.42	Hydrophobic-Pi-Alkyl
22	PHE11	Pi-Orbitals	***VAL6***	Alkyl	4.93	Hydrophobic-Pi-Alkyl
23	PHE17	Pi-Orbitals	***MET12***	Alkyl	5.19	Hydrophobic-Pi-Alkyl
24	PHE17	Pi-Orbitals	***ARG13***	Alkyl	4.25	Hydrophobic-Pi-Alkyl
25	PHE50	Pi-Orbitals	***LEU8***	Alkyl	5.11	Hydrophobic-Pi-Alkyl
26	TYR103	Pi-Orbitals	***ARG13***	Alkyl	5.11	Hydrophobic-Pi-Alkyl
27	TYR163	Pi-Orbitals	***ARG4***	Alkyl	5.28	Hydrophobic-Pi-Alkyl

SP40 peptide residues involved in interactions are in bold and in italic.

**Table 2:** Residues in annexin A2 involved in interactions with the SP40 peptide.

No	H-Donor Residue	Position	H-Acceptor Residue	Position	Distance	Type of Interaction
1	ARG244	HH21	***GLU7***	OE2	2.86	Salt Bridge
2	LYS285	HZ2	***ASP15***	OXT	3.11	Salt Bridge
3	***LYS5***	H25	ASP238	OD1	2.63	Salt Bridge
4	***ARG13***	H26	GLU124	OE1	2.92	Salt Bridge
5	LYS285	NZ	***ASP15***	O	4.34	Electrostatic
6	***ARG3***	NH1	ASP238	OD1	5.51	Electrostatic
7	***ARG3***	NH2	GLU241	OE1	5.26	Electrostatic
8	***LYS5***	NZ	GLU241	OE1	4.29	Electrostatic
9	***ARG13***	NH1	ASP123	OD1	3.57	Electrostatic
10	***ARG13***	NH2	ASP123	OD2	4.28	Electrostatic
11	LYS232	HZ1	***GLN1***	OE1	2.21	Conventional hydrogen bond
12	GLN260	HE21	***GLU7***	OE1	1.81	Conventional hydrogen bond
13	LYS285	HZ3	***PHE14***	O	2.59	Conventional hydrogen bond
14	***ARG4***	H25	LYS309	O	2.71	Conventional hydrogen bond
15	***ARG4***	H25	LYS309	O	1.53	Conventional hydrogen bond
16	***ARG13***	H26	THR122	O	2.50	Conventional hydrogen bond
17	ARG283	HD1	***ARG13***	O	2.96	Carbon hydrogen bond
18	ARG283	HD2	***TYR11***	O	2.11	Carbon hydrogen bond
19	LYS285	HE2	***PHE14***	O	2.56	Carbon hydrogen bond
20	***ARG13***	H26	ASP123	OD1	2.98	Carbon hydrogen bond
21	ASP284	H	***PHE14***	Pi-Orbitals	2.99	Pi-Donor hydrogen bond
22	THR282	O	***PHE14***	Pi-Orbitals	2.77	Pi-Lone Pair
23	LEU267	Alkyl	***MET2***	Alkyl	4.68	Hydrophobic-Alkyl
24	ARG283	Alkyl	***MET12***	Alkyl	4.85	Hydrophobic-Alkyl
25	***LYS5***	Alkyl	LEU240	Alkyl	5.43	Hydrophobic-Alkyl
26	***PHE14***	Pi-Orbitals	ARG283	Alkyl	5.11	Hydrophobic-Pi-Alkyl

SP40 peptide residues involved in interactions are in bold and in italics.

**Table 3:** Residues in SCARB2 involved in interactions with the SP40 peptide.

No	H-Donor Residue	Position	H-Acceptor Residue	Position	Distance	Type of Interaction
1	***LYS5***	H31	ASP194	OD2	1.72	Salt Bridge
2	TYR388	HH	***ASP15***	OD1	2.21	Conventional hydrogen bond
3	***GLN1***	H31	GLU204	OE2	2.21	Conventional hydrogen bond
4	***ARG3***	H31	GLY200	O	1.70	Conventional hydrogen bond
5	***ARG3***	H31	GLY200	O	2.25	Conventional hydrogen bond
6	***ARG4***	H31	SER196	O	2.83	Conventional hydrogen bond
7	***ARG4***	H31	TYR198	O	2.39	Conventional hydrogen bond
8	***THR10***	H32	GLY332	O	2.36	Conventional hydrogen bond
9	***ARG13***	H31	GLN148	OE1	2.72	Conventional hydrogen bond
10	***ARG13***	H32	GLN148	O	2.81	Conventional hydrogen bond
11	***ARG13***	H32	GLN148	OE1	1.64	Conventional hydrogen bond
12	***ARG3***	H31	GLU204	OE1	1.96	Carbon hydrogen bond
13	***ARG3***	H31	TYR203	O	2.61	Carbon hydrogen bond
14	***ARG3***	NH2	PHE199	Pi-Orbitals	4.14	Electrostatic-Pi-Cation
15	GLU145	OE1	***PHE14***	Pi-Orbitals	4.79	Electrostatic-Pi-Anion
16	TRP146	Pi-Orbitals	***PHE9***	Pi-Orbitals	3.70	Hydrophobic-Pi-Pi Stacked
17	TRP146	Pi-Orbitals	***PHE9***	Pi-Orbitals	3.60	Hydrophobic-Pi-Pi Stacked
18	PRO314	Alkyl	***MET12***	Alkyl	5.37	Hydrophobic-Alkyl
19	ILE328	Alkyl	***LEU8***	Alkyl	4.88	Hydrophobic-Alkyl

SP40 peptide residues involved in interactions are in bold and in italics.

**Table 4:** Residues in human tryptophanyl-tRNA synthetase involved in interactions with the SP40 peptide.

No	H-Donor Residue	Position	H-Acceptor Residue	Position	Distance	Type of Interaction
1	LYS154	NZ	***ASP15***	O	4.41	Electrostatic
2	***ARG3***	NH2	GLU151	OE1	4.31	Electrostatic
3	***ARG13***	NH1	ASP302	OD2	5.10	Electrostatic
4	TYR150	HH	***ARG3***	O	2.75	Conventional Hydrogen Bond
5	ASP469	H	***GLU7***	OE2	2.20	Conventional Hydrogen Bond
6	***GLN1***	H30	ARG122	O	2.44	Conventional Hydrogen Bond
7	***GLN1***	H32	ARG122	O	3.02	Conventional Hydrogen Bond
8	***ARG3***	H31	GLU151	O	3.05	Conventional Hydrogen Bond
9	***PHE14***	H32	ASP302	OD2	1.54	Conventional Hydrogen Bond
10	***ASP15***	H32	ASP302	OD2	2.77	Conventional Hydrogen Bond
11	LYS154	*HE1*	***PHE14***	O	2.84	Carbon Hydrogen Bond
12	PHE468	*HA*	***GLU7***	OE2	2.93	Carbon Hydrogen Bond
13	***MET2***	H30	ALA123	O	3.10	Carbon Hydrogen Bond
14	***ARG3***	H31	GLU151	O	2.88	Carbon Hydrogen Bond
15	***ARG13***	H32	ASP302	OD2	2.48	Carbon Hydrogen Bond
16	***LYS5***	NZ	PHE468	Pi-Orbitals	4.46	Electrostatic-Pi-Cation
17	LYS153	Alkyl	***VAL6***	Alkyl	4.77	Hydrophobic-Alkyl
18	***ARG4***	Alkyl	LYS153	Alkyl	5.48	Hydrophobic-Alkyl
19	TYR150	Pi-Orbitals	***ARG3***	Alkyl	4.37	Hydrophobic-Pi-Alkyl
20	HIS472	Pi-Orbitals	***MET12***	Alkyl	4.19	Hydrophobic-Pi-Alkyl
21	***PHE9***	Pi-Orbitals	PRO155	Alkyl	4.10	Hydrophobic-Pi-Alkyl
22	***PHE14***	Pi-Orbitals	LYS154	Alkyl	4.47	Hydrophobic-Pi-Alkyl

SP40 peptide residues involved in interactions are in bold and in italic.

**Table 5:** Comparison of reduction in viral infectivity after antibody treatments against specific receptors in the presence or absence of SP40 peptide.

Antibody Treatment	Inhibition of Viral Infectivity (%)	Inhibition of Viral Infectivity with L-SP40 Peptide (%)
Anti-nucleolin	92.88 ± 1.40	96.28 ± 0.72
Anti-annexin A2	89.55 ± 1.85	92.87 ± 2.06
Anti-PSGL-1	75.91 ± 11.38	88.56 ± 4.38
Anti-vimentin	65.13 ± 10.91	96.46 ± 1.21
Anti-heparan Sulfate	63.16 ± 7.49	98.2 ± 0.68
Anti-galectin-1	58.5 ± 4.25	95.43 ± 1.90
Anti-SCARB2	51.04 ± 1.81	99.54 ± 0.34

PSGL-1: P-selectin glycoprotein ligand-1, SCARB2: human scavenger receptor class B member 2. Inhibition of EV-A71 infection by L-SP40 peptide (100 µM) was 89.55 ± 1.85. Infectivity was determined by plaque formation assay in Rhabdomyosarcoma (RD) cells. SD denotes standard deviations ± SD.

**Table 6:** Description of antibodies used in this study.

Antibody	Immunogen	Concentration µg/mL
Annexin A2	Synthetic peptide within human Annexin A2 aa 20–60.	10
Anti-vimentin	Raised against purified Vimentin	20
Anti-PSGL-1	The antibody epitope was mapped to a site within a consensus tyrosine Sulfation motif of PSGL-1, previously shown to be essential for interaction with P-selectin (and now shown to be essential for recognition of PSGL-1 by L-selectin).	20
Anti-heparan sulphate	Heparan Sulphate Proteoglycan from EHS mouse tumour	20
Anti-gelactin-1	Synthetic peptide. This information is proprietary to Abcam and/or its suppliers	20
Anti-SCARB2	Mouse myeloma cell line NS0-derived recombinant human LIMPII lumenal loop Arg27-Thr432	20
Anti-nucleolin (monoclonal)	Human nucleolin protein from Raji cell extract	20
Anti-nucleolin (polyclonal)	Synthetic peptide conjugated to KLH derived from within residues 1–100 of Human Nucleolin	20

## Supplementary Materials

Table S1: General molecular docking of SP40 peptide with different target cellular receptors using AutoDock4 software, Figure S1: The first five binding pose of SP40 peptide interacting with the receptors in global molecular docking via HPEPDOCK Table S2: Fitness Gold score of local molecular docking of SP40 peptide with different target cellular receptors using GOLD software.

## Sample Availability

Samples such as peptide or antibodies employed in this study were purchased from commercial suppliers.
