# Supplementary material for: Molecular Docking of SP40 Peptide towards Cellular Receptors for Enterovirus 71 (EV-A71)
Source: Molecules. 2021 Oct 30;26(21):6576. doi: 10.3390/molecules26216576 (PMC8587434; doi:10.3390/molecules26216576)
Supplement: Supplementary file 1 [file molecules-26-06576-s001.zip › molecules-1320871-supplementary.pdf]

Supplementary Materials

Article

Molecular docking of SP40 peptide towards cellular receptors for Enterovirus 71 (EV-A71)

Malihe Masomian [1,] *, Salima Lalani [1] and Chit Laa Poh [1,] *

[1] Centre of Virus and Vaccine Research, School of Medical and Life Science, Sunway University, Bandar Sunway, 47500, Selangor, Malaysia; malihem@sunway.edu.my; salima.l@imail.sunway.edu.my; pohcl@sunway.edu.my

* Correspondence: malihem@sunway.edu.my (MM); pohcl@sunway.edu.my (CLP)
Tel.: +603-74918622(Ext 7338)

Table S1: General molecular docking of SP40 peptide with different target cellular receptors using HPEPDOCK software.

Receptors	Model 1	Model 2	Model 3	Model 4	Model 5	Model 6	Model 7	Model 8	Model 9	Model 10
**Nucleolin**	-249.4	-244.6	-238.7	-235.1	-233.1	-228.7	-227.9	-224.4	-223.4	-222.9
**Human cyclophilin**	-231.8	-223.8	-217.3	-212.7	-212.4	-210.1	-209.7	-209.3	-209.1	-207.7
**Human galectin**	-229.2	-198.7	-197.3	-193.7	-188.7	-186.9	-186.3	-186.1	-185.6	-185.0
**Fibronectin**	-227.3	-203.5	-186.4	-186.3	-183.5	-182.0	-181.3	-180.6	-179.9	-179.2
**SCARB2**	-224.2	-215.1	-211.1	-210.2	-202.7	-200.4	-199.6	-199.5	-197.5	-196.6
**DC-SIGN**	-194.9	-189.9	-187.0	-186.0	-183.1	-181.8	-181.7	-181.1	-180.7	-178.6
**Annexin**	-193.4	-179.5	-178.6	-177.6	-177.4	-174.1	-173.6	-170.9	-170.0	-169.4
**Human tryptophanyl-tRNA synthetase**	-191.4	-189.7	-185.2	-185.1	-185.1	-184.4	-180.5	-177.2	-176.6	-176.0
**Vimentin**	-182.5	-180.2	-167.7	-167.5	-159.8	-157.8	-156.7	-156.6	-153.4	-151.4
**Human prohibitin**	-156.1	-152.3	-149.9	-149.5	-146.2	-145.2	-141.9	-140.6	-140.3	-140.1

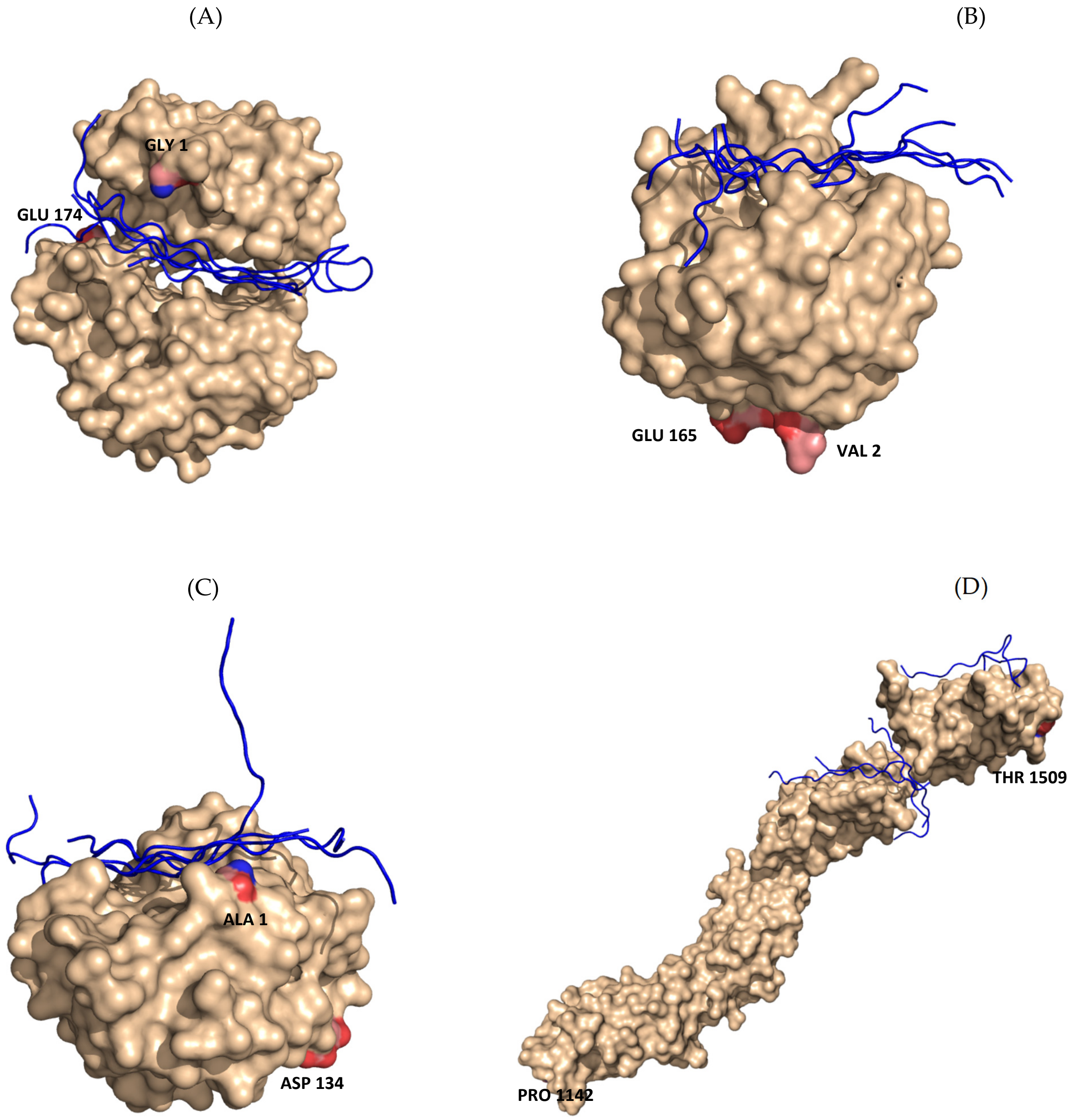

(A)
GLY 1
GLU 174
(B)
GLU 165
VAL 2
(C)
ALA 1
ASP 134
(D)
THR 1509
PRO 1142

(E)

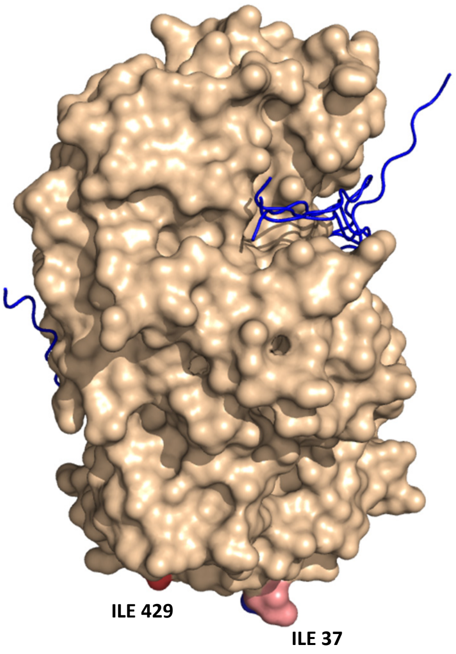

(F)

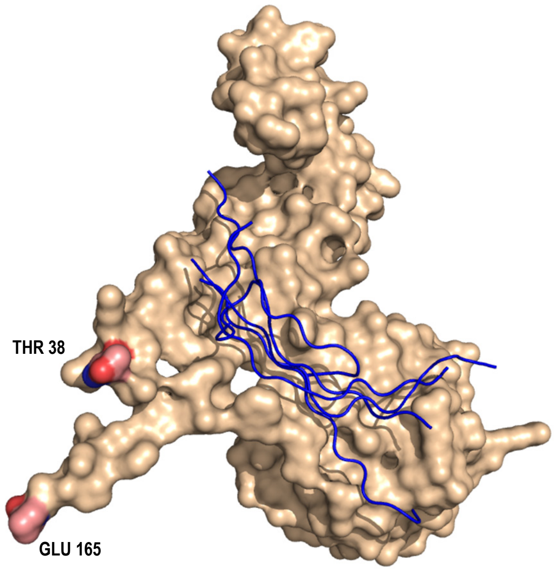

(G)

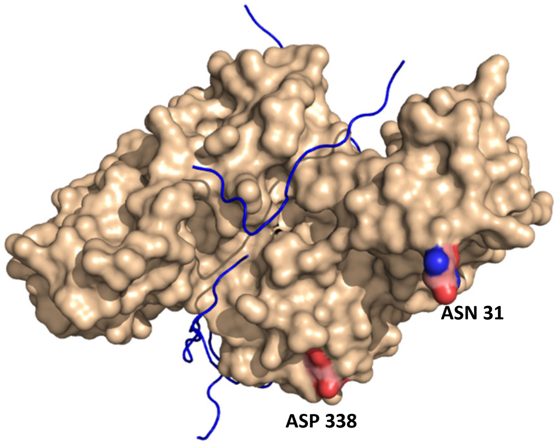

` (H)

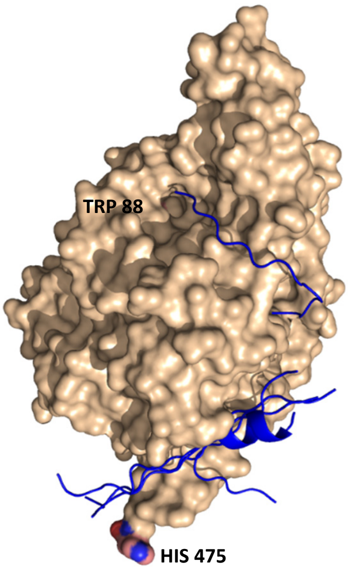

(I)

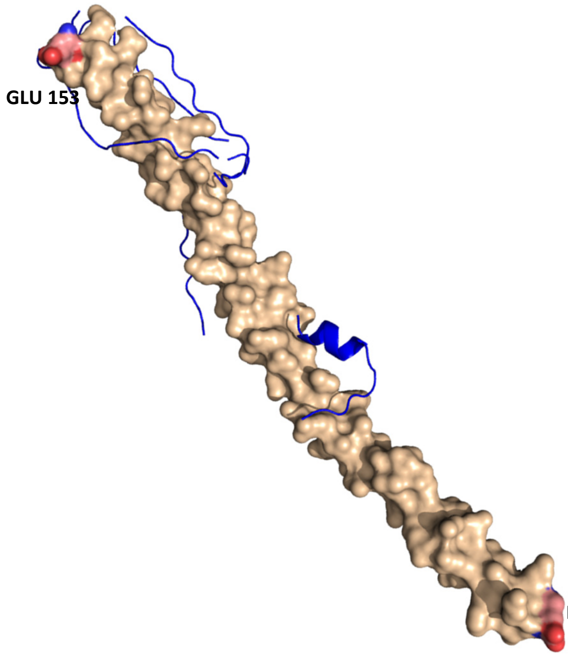

(J)

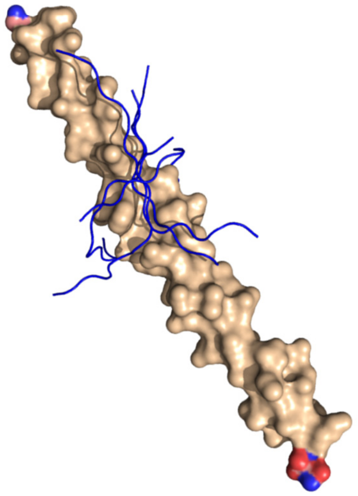

Figure S1: The top- five binding poses of SP40 peptide interacting with the receptors in global molecular docking via HPEPDOCK. The surface of the receptors and SP40 peptides are shown in wheat and blue colors, respectively. A: Nucleolin, B: Human cyclophilin, C: Human galectin, D: Fibronectin, E: SCARB2, F: DC-SIGN, G: Annexin A2, H: Human tryptophanyl-tRNA synthetase l, I: Vimentin and J: Human prohibitin.

Table S2: Fitness Gold score of local molecular docking of SP40 peptide with different target cellular receptors using GOLD software

Receptors	Model 1	Model 2	Model 3	Model 4	Model 5
**Nucleolin**	**125.25**	116.64	101.35	90.92	114.91
**Annexin A2**	97.22	107.62	91.01	**113.48**	104.27
**SCARB2**	94.39	**110.9**	96.55	107.16	87.86
**Vimentin**	79.24	67.92	54.97	**109.19**	65.73
**Human tryptophanyl-tRNA synthetase**	96.75	92.98	100.05	**108.5**	97.49
**Human cyclophilin**	**107.75**	105.87	86.73	84.53	93.33
**Fibronectin**	88.94	82.72	105.53	**107.02**	106.83
**DC-SIGN**	79.87	94.49	89.58	60.3	**105.74**
**Human galectin**	81.06	86.3	98.83	95.46	**99.2**
**Human prohibitin**	54.05	**55.43**	11	0	40.91
